# Using JADES NIRCam photometry to investigate the dependence of stellar mass inferences on the IMF in the early universe

**DOI:** 10.1073/pnas.2317375121

**Published:** 2024-10-08

**Authors:** Charity Woodrum, Marcia Rieke, Zhiyuan Ji, William M. Baker, Rachana Bhatawdekar, Andrew J. Bunker, Stéphane Charlot, Emma Curtis-Lake, Daniel J. Eisenstein, Kevin Hainline, Ryan Hausen, Jakob M. Helton, Raphael E. Hviding, Benjamin D. Johnson, Brant Robertson, Fengwu Sun, Sandro Tacchella, Lily Whitler, Christina C. Williams, Christopher N. A. Willmer

**Affiliations:** ^a^Steward Observatory, University of Arizona, Tucson, AZ 85721; ^b^Kavli Institute for Cosmology, University of Cambridge, Cambridge CB3 0HA, United Kingdom; ^c^Department of Physics, Cavendish Laboratory, University of Cambridge, Cambridge CB3 0HE, United Kingdom; ^d^European Space Agency (ESA), European Space Astronomy Centre (ESAC), Madrid 28692, Spain; ^e^Department of Physics, University of Oxford, Oxford OX1 3RH, United Kingdom; ^f^Sorbonne Université, CNRS, UMR 7095, Institut d’Astrophysique de Paris, Paris 75014, France; ^g^Centre for Astrophysics Research, Department of Physics, Astronomy, and Mathematics, University of Hertfordshire, Hatfield AL10 9AB, United Kingdom; ^h^Center for Astrophysics, Harvard and Smithsonian, Cambridge, MA 02138; ^i^Department of Physics and Astronomy, The Johns Hopkins University, Baltimore, MD 21218; ^j^Max-Planck-Institut für Astronomie, Heidelberg D-69117, Germany; ^k^Department of Astronomy and Astrophysics, University of California, Santa Cruz, CA 95064; ^l^NSF’s National Optical-Infrared Astronomy Research Laboratory, Tucson, AZ 85719

**Keywords:** galaxy evolution, high-redshift galaxies, James Webb Space Telescope, star formation

## Abstract

The detection of numerous and relatively bright galaxies at redshifts *z* > 9 has prompted new investigations into the star-forming properties of high-redshift galaxies. Using local forms of the initial mass function (IMF) to estimate stellar masses of these galaxies from their light output leads to galaxy masses that are at the limit allowed for the state of the Lambda Cold Dark Matter (ΛCDM) Universe at their redshift. We explore how varying the IMF assumed in studies of galaxies in the early universe changes the inferred values for the stellar masses of these galaxies. We infer galaxy properties with the spectral energy distribution (SED) fitting code Prospector using varying IMF parameterizations for a sample of 102 galaxies with photometry from the James Webb Space Telescope, JWST Advanced Deep Extragalactic Survey that are spectroscopically confirmed to be at z>6.7, with additional photometry from the JWST Extragalactic Medium Band Survey for twenty-one of the galaxies. We demonstrate that models with stellar masses reduced by a factor of three or more do not affect the modeled SED.

## Significance

The James Webb Space Telescope (JWST) has enabled the study of the infant universe in unprecedented detail with the hope of revealing how the first galaxies formed and subsequently evolved. If these data were interpreted in the framework of star formation processes in the Milky Way, JWST observations likely contradict cold dark matter theory predictions and would force a reassessment of basic physics. Using a sample of distant galaxies with high-quality photometry and spectroscopically confirmed distances, we investigate how changing star formation parameters avoids such a contradiction with galaxy age and stellar mass being traded against each other to match observed galaxy properties. The cold dark matter paradigm remains consistent with observations.

The initial deep surveys using the James Webb Space Telescope (JWST) have changed our view of the high redshift Universe and have posed new challenges to our understanding galaxy formation. A common finding from the first deep imagery is an excess of bright galaxies as compared to most theoretical expectations for redshifts above 8 in ref. 1 with a summary in ref. 2. Many mechanisms might explain this excess as discussed in ref. 3 such as enhanced star formation efficiency, little or no dust attenuation at the highest redshifts, and changes in the initial mass function (IMF). The IMF is a key issue in understanding these bright galaxies since it is central to translating an observed brightness into mass, a difficult calculation for these objects seen only ∼300 to 600 My after the Big Bang. The IMF and whether it has a universal shape has been a subject of long-standing debate (see ref. 4 for a review). Factors that influence the shape of the IMF and whether it is weighted toward high mass stars (top-heavy) or low mass stars (bottom-heavy) include gas temperature, gas density, turbulence, magnetic fields, and metallicity. Stellar binarity can also change the translation of light into mass (5).

Star formation in galaxies at z ∼ 10 must be quite different than local star formation simply because the state of the gas in galaxies at these early times must be different than locally. The dust content and metallicity of the gas are different with lower chemical enrichment (6), As discussed below, the gas is warmer and also very likely denser. The star formation rates are likely higher with the study of low redshift galaxies (z ∼ 0.25) suggesting a correlation where galaxies with higher star formation rates have more top-heavy IMFs (7), a choice favored in some models (8). The lower metallicity in high redshift galaxies may result in a more top-heavy IMF (9). The temperature of the cosmic microwave background is significantly higher at these early times, though this may not be as significant as the increase in dust temperature due to PopII star formation (meaning the second generation of stars to form) and the presence of silicate dust (10). Ref. 11 presented evidence that for massive elliptical galaxies, even by z ∼ 1, the IMF was already weighted more toward massive stars than the local IMF, but note that ref. 12 reports on a galaxy at z ∼ 2 that may have a bottom heavy IMF based on its mass derived from gravitational lensing.

Refs. 13 and 14 discuss magneto-hydrodynamic models of star formation and the IMF with ref. 13 using metallicities $Z_{⊙}\geq 0.01$ and with the gas temperature equal to the dust temperature. Ref. 14 considers conditions likely most similar to those for the highest redshift galaxies (eg. *z* > 10) using metallicities $Z_{⊙}\leq 0.001$ and conclude that a top-heavy IMF prevails in these earliest galaxies. Ref. 15 shows that present-day massive galaxies show spectral features indicative of a bottom heavy IMF. However, as reviewed by ref. 16, other techniques yielding mass estimates for local massive galaxies do not agree on the need for a bottom heavy IMF. Another possibility is an IMF that changes with the progression of star formation in a galaxy with the first epoch of star formation weighted toward high mass stars and later star formation weighted toward low mass stars because the initial star formation alters the interstellar medium (see ref. 17 for a discussion of this possibility).

Other evidence bearing on star formation modes at the earliest epochs comes from unexpected elemental abundances such as the high nitrogen abundance observed in GNz11 ( 18), in GHZ2 ( 19) and galaxies in ref. 20 and attributed to a top heavy IMF by ref. 21. Discovery of a galaxy with large amounts of carbonaceous dust at an age of ∼600 My, ref. 22, a type and quantity of dust that is difficult to produce on this time scale, provides another example of surprising abundances. One possible explanation is dust produced in supernovae from high mass stars rather than the asymptotic giant branch (AGB) star route which suggests more high mass stars.

In this paper, we use the deep multi-band photometry from the JWST Advanced Deep Extragalactic Survey (JADES) to explore how much the IMF can be perturbed without affecting the match to the observed spectral energy distribution (SED) for a single burst. This study is similar to ones reported in refs. 23 and 24, but differs in analyzing a sample of 102 galaxies from z = 6.7 to z = 13.2, which have measured spectroscopic redshifts and high-quality photometry. We fit the SEDs for these sources using Prospector (v1.1.0, ref. 25) with modified IMFs similar to that presented in ref. 24. Several recent papers (23, 26, 27) explore how a temperature-dependent IMF results in a top-heavy IMF and how this change to the IMF changes aspects of deriving galaxy properties by template fitting. Our study differs from ref. 23 as we use Prospector to ensure that a galaxy model can be no older than the age of the Universe at the redshift of the galaxy being fit, and to include nebular emission lines which can be strong enough to influence the flux measured through a broad filter. We find that a top-heavy IMF is consistent with the observed SEDs which is not surprising as the low-mass end of the IMF provides minimal contributions to the luminosity even locally but we have done this in the context of fitting observed galaxies. This work is not aimed at deriving the IMF for these galaxies but rather showing that there is a straightforward possible solution to the apparently high masses inferred for similar galaxies. Most likely, a combination of factors controlling the star formation process in the early universe will be required for a complete explanation. See ref. 28 for a discussion of how a series of star formation bursts can further complicate stellar mass determination. We adopt the standard flat ΛCDM cosmology from Planck18 with *H*_0_ = 67.4 km/s/Mpc and $\Omega_{m}$ = 0.315 (29).

## Data

We utilize deep space-based imaging from JWST with at least eight Near Infrared Camera (NIRCam) photometric bands in the GOODS-S field. The sources all have spectroscopic redshifts measured either as part of the JADES program using the Near Infrared Spectrograph (NIRSpec), refs. 18 and 30, or as part of the JWST First Reionization Epoch Spectroscopically Complete Observations (FRESCO) (Program ID 1895, PI P. Oesch) program using grisms in NIRCam with redshifts and tabulated in *SI Appendix*. The JWST/NIRCam photometry is part of the JWST Advanced Deep Extragalactic Survey (31, 32). The imaging data include F090W, F115W, F150W, F200W, F277W, F356W, F410M, and F444W for the main sample. For a subset of the sample, F182M, F210M, F430M, F460M, and F480M images are also available as part of the JWST Extragalactic Medium-band Survey (JEMS; ref. 33). As part of the JADES imaging some of these galaxies also have F335M photometry. *SI Appendix* tabulates the redshifts and photometry for the galaxies used in this study.

### Sample Selection.

**Fig. 1.**
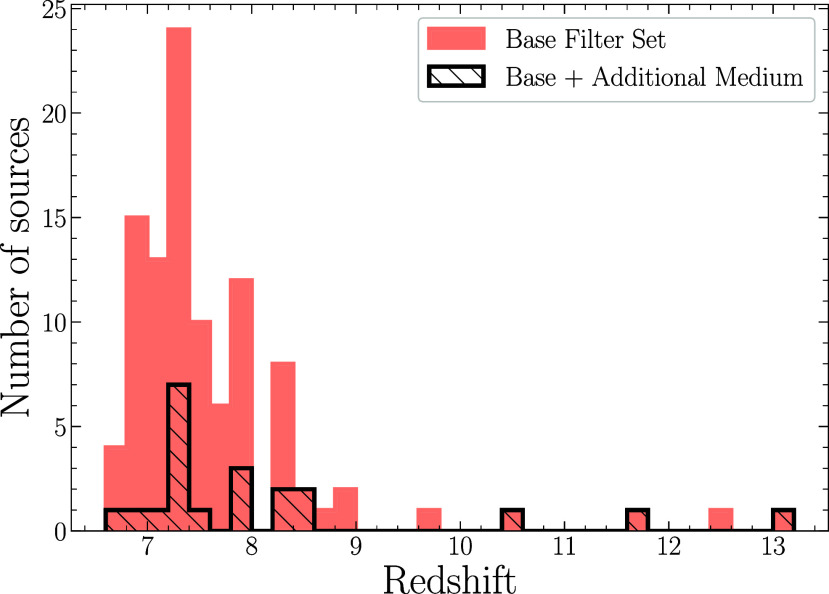
The distribution of spectroscopic redshifts for our sample of 102 galaxies from JADES including twenty-one galaxies that have additional medium band photometry from JEMS.

The primary sample consists of 102 galaxies from the JADES that have confirmed spectroscopic redshifts. Ninety galaxies have FRESCO redshifts, eleven have NIRSpec redshifts, and one galaxy has both. To be included in this sample, the galaxies were also required to have a signal-to-noise ratio greater than five in at least five photometric bands. We select galaxies with z>6.7, the lowest redshift at which λ5,007Å [OIII] would be observable in the FRESCO data, which is the main source of our redshifts. This redshift range means that few objects would be detected in F090W because of attenuation in the intergalactic medium (34). Any objects with a large (>0.5) difference between the spectroscopic redshift and the photometric redshift (35) were inspected visually and one object was rejected due to overlapping components. Twenty-one galaxies also have imagery in the medium bands as mentioned above. Results do not differ between the samples with and without JEMS data. The F335M data were acquired as part of the JADES program while the other filters were observed as part of the JEMS program, ref. 33. Fig. 1 shows the distribution of spectroscopic redshifts for our sample. Because of the reliance on FRESCO redshifts, most of the sources in our sample have strong emission lines with the quiescent galaxy found by ref. 36 as the only object which definitively does not have emission lines. This selection potentially biases our galaxies toward those with vigorous star formation, but currently it is unknown what fraction of z>6.7 galaxies have strong star formation ( 37). Whether the highest redshift portion of our sample, galaxies at z>9.5, has emission lines is indeterminate as λ5,007Å [OIII] is redshifted beyond the longest wavelength detected by near-infrared instruments, and detection of shorter wavelength lines which are much weaker requires much higher signal-to-noise than is typically available now. Ly-*α* which might be strong is absorbed by the intergalactic medium (e.g., ref. 34).

Photometry was extracted following the procedures outlined in ref. 32. The Prospector analysis used fluxes derived from images convolved with the Point Spread Function (PSF) for the F444W filter (the PSFs for F460M and F480M are marginally larger but this difference was judged to be insignificant for the analyses here as the Kron radii used are typically 40% larger than the 80% encircled energy radii for F444M, F460M, and F480M so ignoring the PSF differences at the longest wavelength results in less than a 2% flux difference). As described in ref. 32, a Kron radius was determined for each source and fluxes measured for the area defined by the Kron radius. These steps ensure that the same spatial fraction of a galaxy is used across the entire NIRCam wavelength range.

## Stellar Population Modeling

We fit the photometry with the Prospector (v.1.1.0; ref. 25) inference framework. Prospector uses the Flexible Stellar Population Synthesis code (FSPS; ref. 38) via python-FSPS ( 39) and Cloudy modeling code for nebular emission, and does not use templates but rather computes SEDs directly. The posterior distributions are sampled using the dynamic nested sampling code dynesty, ref. 40.

We use the same physical model as in ref. 41 following the methodology of ref. 42, with differing IMF prescriptions described in the next section. In brief, the redshifts are fixed at the spectroscopic redshifts shown in Fig. 1. We employ the MESA Isochrones and Stellar Tracks (MIST) stellar evolutionary tracks and isochrones, refs. 43 and 44, which utilizes the Modules for Experiments in Stellar Astrophysics (MESA) stellar evolution package, refs. 45–48. We use Medium-resolution Isaac Newton Telescope Library of Empirical Spectra (MILES) for the stellar spectral library, refs. 49 and 50. The stellar metallicity, log($Z_{*}/Z_{⊙}$), was allowed to range from −2.0 to 0.19. The gas metallicity, log($Z_{\mathrm{gas}}/Z_{⊙}$), was allowed to range from −2.0 to 0.5. The intergalactic medium (IGM) absorption is modeled after (34), where the overall scaling of the IGM attenuation is a free parameter. For dust attenuation, we assume a flexible attenuation curve with the ultraviolet (UV) bump tied to the slope of the curve, ref. 51, and a two-component dust model, ref. 52. The nebular emission is based on Cloudy model grids, ref. 53, and includes both nebular continuum and emission line components. The ionization parameter, log(U), was allowed to range from −4 to −1. For the star formation history (SFH), we use a nonparametric model with the standard continuity prior with six distinct time bins of constant star formation. The bins span from the time of observation to an adopted formation redshift of $z_{\mathrm{form}}=20$. The two most recent age bins are fixed at 0 to 30 Myr and 30 to 100 Myr in lookback time in the galaxy’s reference frame. The last bin is fixed between 0.85$T_{\mathrm{univ}}$ and $T_{\mathrm{univ}}$, where $T_{\mathrm{univ}}$ is the age of the Universe at the galaxy’s spectroscopic redshift, assuming $z_{\mathrm{form}}=20$. The remaining three bins are spaced evenly in logarithmic time. Changing the SFH prior can also lead to changes in the inferred stellar mass, as has been investigated by refs. 42, 54, and 55, however our focus in this paper is on the IMF. Changing the SFH prior mainly affects the amount of mass converted into stars as a function of time with only secondary effects on the total stellar mass for the galaxies at the redshifts in our sample. We note that use of the continuity SFH prior as used in ref. 42 does not bias our results as this prior yields stellar masses in the middle of the range for the priors they tested.

## IMF

One of the most commonly used parameterizations for the IMF is modeled by a lognormal distribution with a characteristic mass, $m_{c}$, ( 56, hereafter C03). In Milky Way studies, another formulation of the IMF uses a broken power law ( 57) where the mass breakpoints could be dependent on temperature or other environmental factors. van Dokkum, ( 11, hereafter V08) introduced a slightly modified form of the C03 IMF which allows for a varying $m_{c}$, where $m_{c}=0.08M_{⊙}$ is almost identical to the C03 IMF. We use this formulation for computational convenience. The functional form of V08 is:


[1] $$\xi (m)=\{\begin{array}{ll} A_{l}{\left(0.5n_{c}m_{c}\right)}^{-x}\text{exp}\left[\text{-}\frac{{\left(\text{log}\;\text{m-log}\;{\text{m}}_{\text{c}}\right)}^{\text{2}}}{\text{2}\sigma^{\text{2}}}\right], & m\leq n_{c}m_{c},\\ A_{h}m^{-x}, & m>n_{c}m_{c}, \end{array}$$

with $A_{l}=0.140$, $n_{c}=25$, *σ* = 0.69, $A_{h}=0.0443$, and x = 1.3 with x referred to as the slope of the IMF. This formulation of the IMF allows the characteristic turnover mass, where the IMF begins to decline, to vary with redshift.

**Fig. 2.**
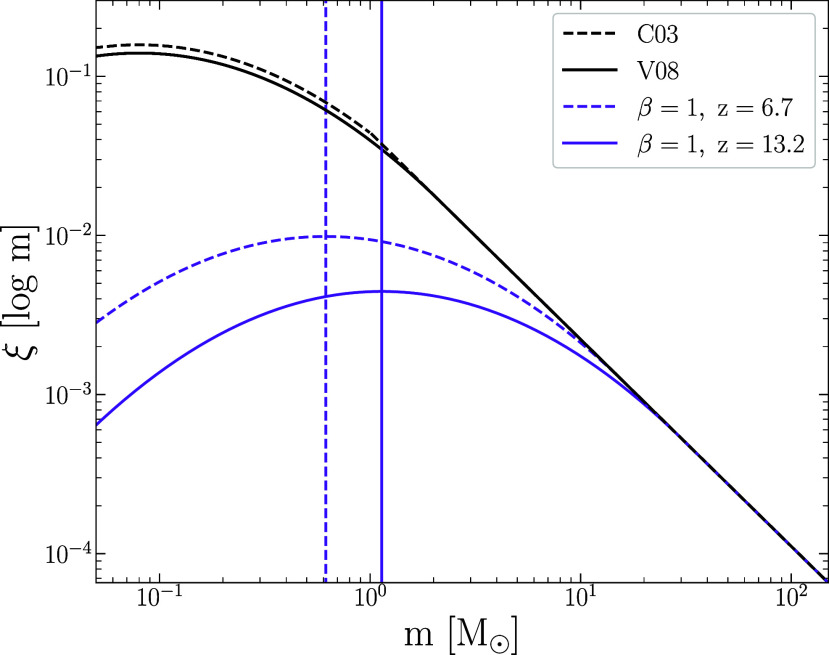
The C03 IMF is shown as a dashed black line compared to the slightly modified form from V08 with $m_{c}=0.08M_{⊙}$ shown as a solid black line. The V08 IMFs with a redshift-dependent characteristic mass, $m_{c}∼{\left(1+z\right)}^{\beta }$, for the minimum and maximum redshifts considered in this paper are shown in purple. The vertical lines indicate the characteristic mass for redshift z = 6.7 (dashed purple line) and for redshift z = 13.2 (solid purple line). These IMFs use *β* = 1.0.

The characteristic mass, $m_{c}$, may change with the temperature of the interstellar medium (ISM). If we assume the ISM temperature of galaxies scales with the temperature of the CMB and purely based on a Jeans argument (58), then $m_{c}∼{\left(T_{\mathrm{ISM}}\right)}^{1.5}∼{\left(T_{\mathrm{CMB}}\right)}^{1.5}∼{\left(1+z\right)}^{1.5}$. Other studies have suggested different scale factors. For example, ref. 59 showed that $m_{c}∼\left(1+\text{z}\right)$ and ref. 60 argue that $m_{c}∼{\left(1+\text{z}\right)}^{2}$. Ref. 61 use Atacama Large Millimeter Array (ALMA) data to show that the dust temperature over the range up to z ∼ 7 increases as ${\left(1+z\right)}^{0.42}$ which supports an increasing value for $m_{c}$. The functional form of the $m_{c}$ to z relation will be more complicated than just the relation for dust temperature because of other factors, such as gas density, which also play a role in setting the mass of collapsing clouds. Various factors may also affect the fragmentation of the star-forming clouds. We modify the V08 IMF by making $m_{c}$ proportional to ${\left(1+\text{z}\right)}^{\beta }$, where *β* = 1, 1.5, and 2, which renders the IMF redshift-dependent. In addition, we use the V08 IMF with *β* = 1.5 and apply a lower limit to the IMF mass. The default lower limit is $M_{\mathrm{limit}}=0.08M_{⊙}$ which we change to $M_{\mathrm{limit}}=1M_{⊙}\;\mathrm{and}\;3M_{⊙}$. We note that the default upper limit on the IMF mass is 120$M_{⊙}$, which we keep unchanged. The effect of changing the upper mass limit would likely result is similar mass estimates but with younger ages. Fig. 2 compares one of our modified IMFs at two redshifts to the original V08 version of the C03 IMF. Our intent is not to prove that one or another of these modifications is the correct IMF but rather to show quantitatively how much the inferred mass changes. The key spectroscopic features that reveal the presence of low mass stars, Na and Wing-Ford bands, are too weak to be detected in galaxies as faint as those studied here (15).

## Results

In this section, we present the inferred physical properties of galaxies in our sample with differing IMF parameterizations described in the previous section. All values are reported as the median, with uncertainties as the 16th and 84th percentiles of the posterior probability. For the quiescent galaxy in our sample, we check whether our fitting results are consistent with those listed in ref. 36. We compare inferred values from our model with the C03 IMF parameterization and their model with the same SFH prior used in this work (the standard continuity prior). We find that all of the inferred parameters are consistent with each other within uncertainties. The inferred Prospector properties are included in *SI Appendix*.

**Fig. 3.**
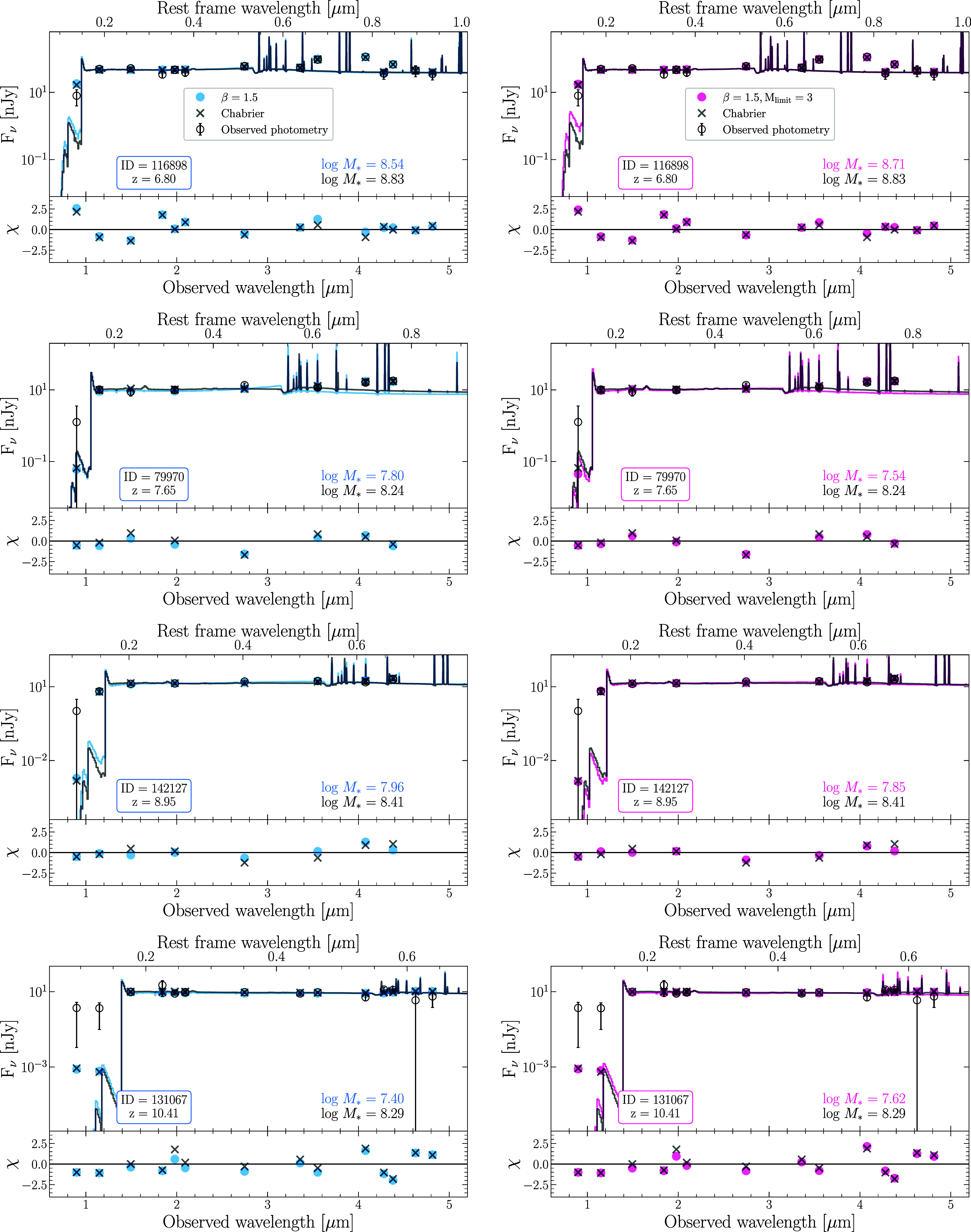
A selection of best-fit Prospector SED models for C03 shown in black and for V08 with *β* = 1.5 shown in blue in the *Left* column and with *β* = 1.5 and $M_{\mathrm{limit}}=3$ in pink in the *Right* column. The smaller panels at the *Bottom* show *χ*, defined as $(F_{\mathrm{model}}-F_{\mathrm{obs}})/\sigma$, which are nearly identical between models. Therefore, our varying IMF parameterizations produce similar best-fit SED models. The vertical scaling has been set to show detail in the SEDs at the expense of clipping the heights of the emission lines.

In Fig. 3, we show examples of the best-fit SEDs for the C03 models compared to the V08 models with *β* = 1.5 and also with *β* = 1.5 and $M_{limit=3}$. The residuals, defined as $\chi =\left(F_{\mathrm{model}}-F_{\mathrm{obs}}\right)/\sigma_{\mathrm{obs}}$, are centered around 0 and show that the data are well fit by the model. In addition, the residuals among the different models are nearly identical.

**Fig. 4.**
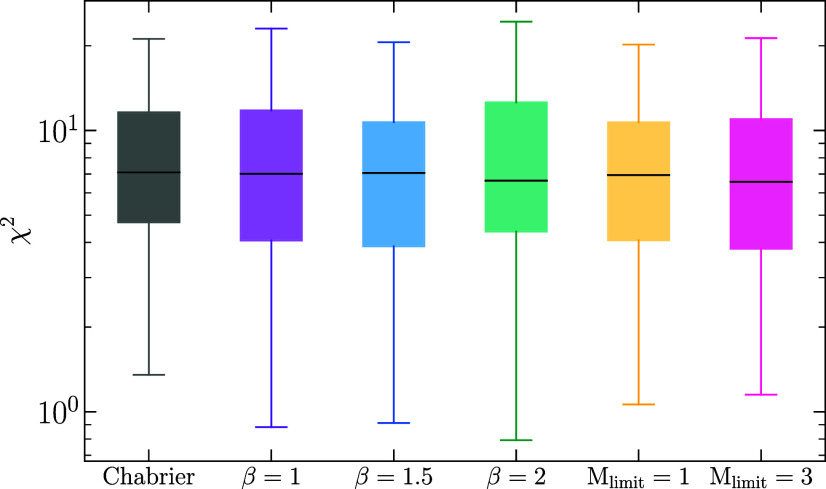
Distributions of the $\chi^{2}$ statistic for each of the different IMF parameterizations. The box extends from the first quartile to the third quartile with the whiskers showing 1.5 times the interquartile range and a line at the median. The data are well fit by all six of the models. In addition, the $\chi^{2}$ statistic is not significantly different between models. Therefore, changing the IMF parameterization does not significantly change the best-fit SED model.

To determine whether the data are better fit by one model over the other, we calculate the $\chi^{2}$ statistic using the best-fit model photometry as $\chi^{2}=\sum {\left(F_{\mathrm{model}}-F_{\mathrm{obs}}\right)}^{2}/\sigma_{\mathrm{obs}}^{2}$, where $F_{\mathrm{model}}$ and $F_{\mathrm{obs}}$ are the observed and model fluxes, respectively, and $\sigma_{\mathrm{obs}}$ is the observed photometric uncertainty; see Fig. 4. We find that the data are well fit by all six of the models. In addition, the $\chi^{2}$ statistic is not significantly different between models. Therefore, changing the IMF parameterization results in model fits that match the observed SEDs equally well.

**Fig. 5.**
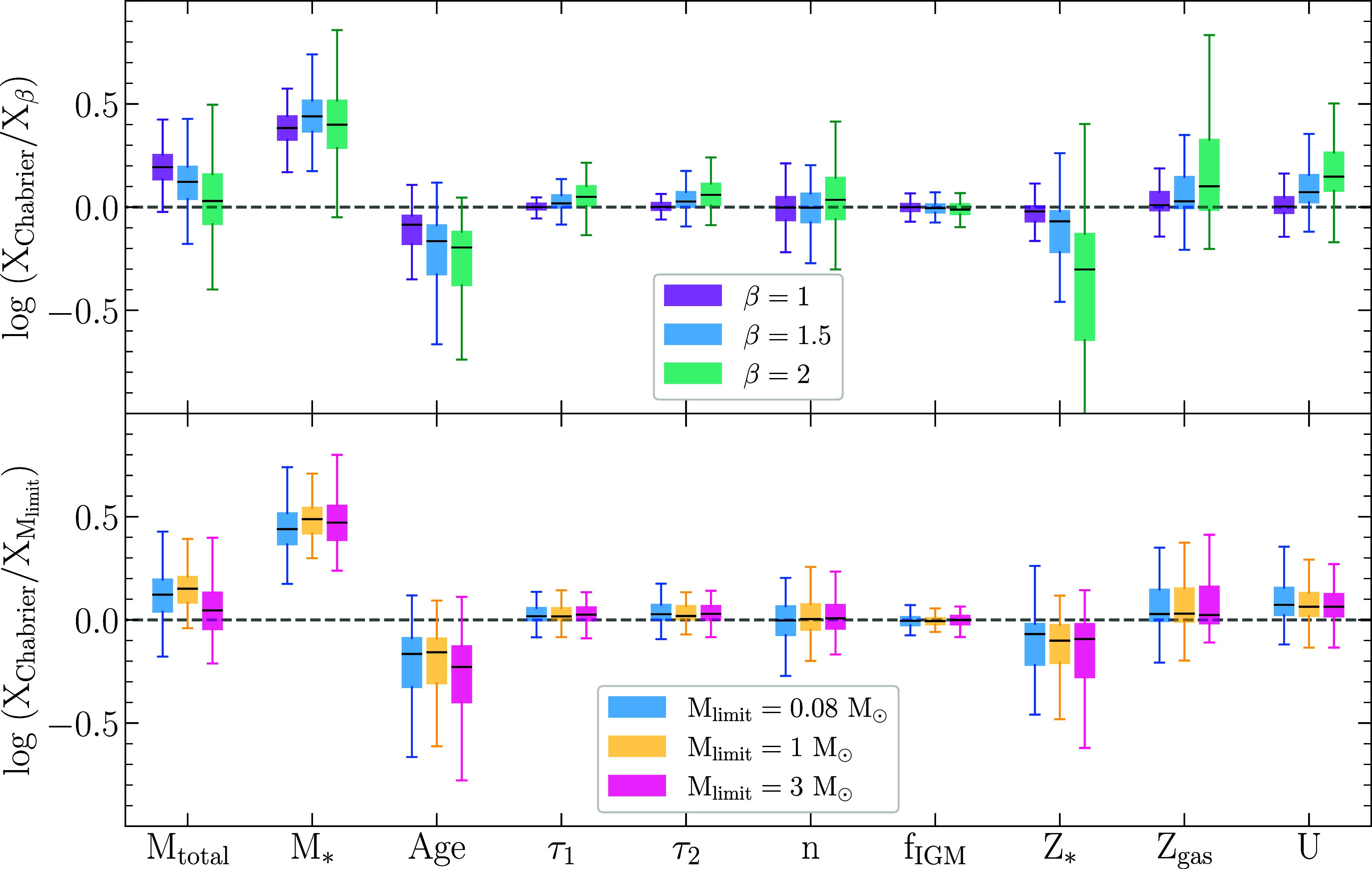
Distributions of the differences between the parameters inferred with our varying IMF parameterizations. The box and whisker ranges are the same as in Fig. 4. The inferred parameters shown here include the total formed mass ($M_{\mathrm{total}}$), the surviving stellar mass ($M_{*}$), the mass-weighted age, the dust optical depth for newly formed stars (*τ*_1_), the diffuse dust optical depth (*τ*_2_), the power-law modifier to the shape of the dust attenuation curve (n), the factor used to scale the IGM attenuation curve ($f_{\mathrm{IGM}}$), the stellar metallicity ($Z_{*}$), the gas-phase metallicity ($Z_{\mathrm{gas}}$), and the ionization parameter for nebular emission (U). The models with varying *lower* mass limits all use *β* = 1.5. Compared to the values inferred using V08 with varying scale factors for $m_{c}$ and varying *lower* limits on the IMF mass, the inferred C03 values are ≈0.1 to 0.2 dex higher for the total formed mass ($M_{\mathrm{total}}$), ≈0.4 to 0.5 dex higher for the surviving stellar mass ($M_{*}$), and ≈0.1 to 0.2 dex*lower* for the mass-weighted age.

**Fig. 6.**
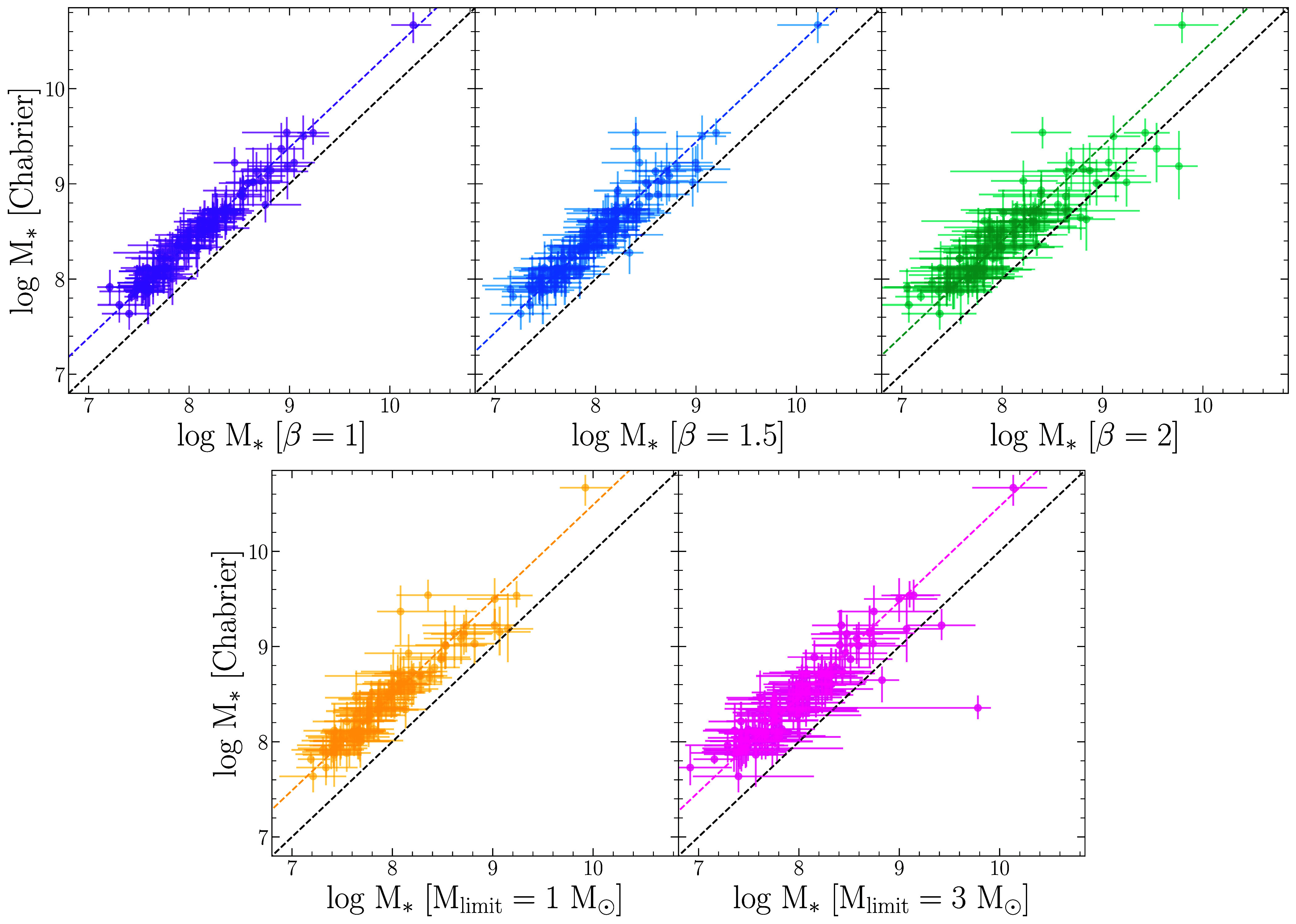
Comparison of stellar masses in units of $M_{⊙}$ determined using a C03 IMF with varying versions of the V08 IMF. For the latter, we let the characteristic mass ($m_{c}$) scale by ${\left(1+z\right)}^{\beta }$. In addition, we place *lower* limits on the IMF mass ($M_{\mathrm{limit}}$). The median offset is shown as a colored dashed line for the different IMF parameterizations.

Next, we compare the distributions of the differences between the inferred galaxy properties using the C03 model and the varying V08 models; see Fig. 5. The inferred parameters include the total formed mass ($M_{\mathrm{total}}$), the surviving stellar mass ($M_{*}$), the mass-weighted age, the birth-cloud dust attenuation (*τ*_1_), the diffuse dust attenuation (*τ*_2_), the power-law modifier to the shape of the dust attenuation curve (n), the factor used to scale the IGM attenuation curve ($f_{\mathrm{IGM}}$), the stellar metallicity ($Z_{*}$), the gas-phase metallicity ($Z_{\mathrm{gas}}$), and the ionization parameter for nebular emission (U). Compared to the values inferred using V08 with varying scale factors for $m_{c}$ and varying lower limits on the IMF masses, the inferred median C03 values are ≈0.1 to 0.2 dex higher for the total formed mass ($M_{\mathrm{total}}$), ≈0.4 to 0.5 dex higher for the surviving stellar mass ($M_{*}$), and ≈0.1 to 0.2 dex lower for the mass-weighted age. The most significant differences are between the stellar masses, which we highlight in Fig. 6 and we list the median offsets in Table 1.

**Table 1.** M_∗_ offsets

IMF	Mass reduction factor
*β* = 1	2.4
*β* = 1.5	2.8
*β* = 2	2.5
$M_{\mathrm{limit}}=1M_{⊙}$	3.1
$M_{\mathrm{limit}}=3M_{⊙}$	3.0

The median offset between stellar masses inferred from the C03 parameterization and the differing V08 parameterizations, shown as dashed lines in Fig. 6.

In summary, varying the IMF parameterization results in SED models that are not substantially different from one another. However, their stellar masses can differ significantly, with over three times smaller inferred stellar masses than for the commonly used C03 model. In addition, the mass-weighted ages are lower for the C03 model, meaning that the C03 model infers SFHs that form larger masses over a shorter amount of time. As mentioned in the introduction, a variety of factors can influence stellar mass estimates derived from SED fitting to observed fluxes. This study is confined to examining how much plausible changes to the IMF change the derived masses, and the factor of three reduction found here, Table 1, would reduce the tension with the allowed amount of stellar mass in ΛCDM models.

## Discussion

Our results indicate that the changes in the IMF that are likely for high redshift star formation can reduce the stellar mass inferred from galaxy photometry by as much as a factor of three as compared to the mass inferred from use of the local C03 IMF. A similar conclusion is reached by refs. 23 and 24.

Ref. 62 studied a sample of Cosmic Evolution Early Release Science (CEERS) galaxies with Mid-infrared Instrument (MIRI) photometry at 5.6 and 7.7 μm which they combined with Hubble Space Telescope (HST) and Infrared Array Camera (IRAC) measurements at 3.6 and 4.5 μm. They found similar reductions in the mass required to fit the SEDs when the MIRI data are included as we find by changing the IMF. Another study (63) looked at using MIRI data to derive galaxy masses and also finds that inclusion of MIRI data in the fitting reduces the required mass but may also require higher star formation efficiency than seen locally. Ref. 64 combines JADES NIRCam data with MIRI and ALMA data to study extremely red galaxies and find reductions in the required mass somewhat larger than found here. They also cite work in preparation considering galaxies with more normal colors where the addition of MIRI data makes little difference. Two of the galaxies in our sample (126594 with z = 7.95 and 219000 with z = 6.81) appear in the ref. 64 sample. Our mass derived without use of MIRI data for 126594 is 0.3 dex lower than their value derived including MIRI data. Our value for 219000 which is quite red is 0.67 dex higher than their value. MIRI data do not necessarily reduce the required mass for galaxies, but the MIRI data are helpful in modeling red galaxies and for identifying active galactic nuclei (eg. refs. 64 and 65).

The galaxies used in this study all have spectroscopic redshifts so there are no uncertainties on the light travel time from them. The redshift interval from z ∼ 13.2 to z ∼ 6.7 corresponds to ages of 320 My and 804 My after the Big Bang, respectively. In terms of stellar evolution, these ages correspond to changes in main sequence turn-off ranging from O and B stars to F stars depending on when the stars first formed. The picture that is developing for these high redshift objects is one with strong on-going star formation as evidenced by the strong emission lines detected, and which are present in nearly all of the galaxies in our sample. The output of high mass stars capable of ionizing the ISM completely hides the low mass end of the mass function so it is not surprising that our Prospector models are so insensitive to the parameterization of the IMF. What is clear is that the high redshift galaxy population being discovered in JWST data is more luminous than expected, (e.g., refs. 66–68), but not necessarily more massive. Because of the many reasons for the high redshift IMF to differ from C03, this component of minimizing the tension with ΛCDM needs to be taken into account. However, a complete understanding of the SFHs of the galaxies at early times awaits more detailed spectroscopy. The solution to measuring galaxy masses accurately will require high spectral resolution data that can be used for measurement of dynamical masses although such data will only provide upper limits on the stellar mass.

## Supplementary Material

Appendix 01 (PDF)

Dataset S01 (XLSX)

## Data Availability

All study data are included in the article and/or supporting information.
