# Supplementary material for: Using JADES NIRCam photometry to investigate the dependence of stellar mass inferences on the IMF in the early universe
Source: Proc Natl Acad Sci U S A. 2024 Oct 8;121(42):e2317375121. doi: 10.1073/pnas.2317375121 (PMC11494360; doi:10.1073/pnas.2317375121)
Supplement: Supplementary file 1 — Appendix 01 (PDF) [file pnas.2317375121.sapp.pdf]

PNAS

Supporting Information for

JADES: Using NIRCam Photometry to Investigate the Dependence of Stellar Mass Inferences on the IMF in the Early Universe

Charity Woodrum, Marcia Rieke, Zhiyuan Ji, William M. Baker, Rachana Bhatawdekar, Andrew J. Bunker, Stéphane Charlot, Emma Curtis-Lake, Daniel J. Eisenstein, Kevin Hainline, Ryan Hausen, Jakob M. Helton, Raphael E. Hviding, Benjamin D. Johnson, Brant Robertson, Fengwu Sun, Sandro Tacchella, Lily Whitler, Christina C. Williams, and Christopher N.A. Willmer

Marcia Rieke
E-mail: mrieke@as.arizona.edu

This PDF file includes:

Supporting text
Table S1
SI References

Supporting Information Text

Multi-band photometry and `Prospector` output for 102 galaxies are presented in an excel spreadsheet. One tab presents the `Prospector` output and a second tab labelled "Photometric Data" presents the fluxes for each galaxy in nJy in a Kron-radius size aperture for all filters used. Ref. (1) describes the measurement of these fluxes. The filters included in the spreadsheet for all galaxies include F090W. F115W, F150W, F200W, F277W, F356W, F410M, and F444W. Those galaxies which also have measurements from JEMS have photometry in the filters F182M, F210M, F335M, F430M, F460M. and F480M. Note that the filter designations indicate the center wavelength as 100x wavelength in microns and W indicates $\lambda/\delta\lambda = 4$ and M indicates $\lambda/\delta\lambda \sim 10$.

The `Prospector` output tabulated in the spreadsheet includes the JADES identification number which can be used to find the galaxies in the JADES photometric catalog available from the Space Telescope Science Institute High Level Science Products (HLSP) at the Mikulski Archive for Space Telescopes (MAST). The other entries on the speadsheet include right ascension, declination, spectroscopic and photometric redshifts, and redshift source. Table S1 lists the `Prospector`-specific output parameters. As described in the main body of the paper, the IMF used is shown in this equation:

$$\xi(m) = \begin{cases} A_l(0.5n_c m_c)^{-x} \exp\left[-\frac{(\log\ \mathrm{m} - \log\ \mathrm{m_c})^2}{2\sigma^2}\right], & m \le n_c m_c, \\ A_h m^{-x}, & m > n_c m_c \end{cases} \qquad [1]$$

with $m_c \propto (1+\mathrm{z})^{\beta}$, β=1, 1.5 or 2, $A_l = 0.140$, $n_c = 25$, $\sigma = 0.69$, $A_h = 0.0443$, and x=1.3

Table S1. `Prospector` output parameters

Parameter	Spreadsheet Column Label
Total Mass (IMF with $\beta = 1$)	$log(M_{total})$
Remaining Stellar Mass (Chabrier (2) IMF)	$log(M_{star})$ (C03)
Remaining Stellar Mass (IMF with $\beta = 1.0$	$log(M_{star})$ ($\beta = 1.0$)
Remaining Stellar Mass (IMF with $\beta = 1.5$ Lower mass limit = 3 $M_\odot$	$log(M_{star})$ ($\beta = 1.0$) $M_{limit} = 3$
Mass weighted age of the galaxy in years	log(age)
Dust optical depth to newly formed stars	τ_1
Dust optical depth affecting all stars	τ_2
Modifier for the dust attenuation power law	n
Intergalactic medium attenuation curve scale factor	f_{IGM}
Stellar metallicity	Z_*
Gas metallicity	Z_{gas}
Nebular emission ionization parameter	U

References

1. MJ Rieke, et al., JADES Initial Data Release for the Hubble Ultra Deep Field: Revealing the Faint Infrared Sky with Deep JWST NIRCam Imaging. *ApJS* **269**, 16 (2023).
2. G Chabrier, Galactic Stellar and Substellar Initial Mass Function. *PASP* **115**, 763–795 (2003).
